# Relationship between bite force, bruxism, and fractures of teeth and dental restorations

**DOI:** 10.1038/s41598-025-07772-2

**Published:** 2025-07-02

**Authors:** Bruno Ramos Chrcanovic, Tom Bergengren, Nikola Stanisic, Sahar Sohrabi, Christel Larsson, Peter Svensson, Birgitta Häggman-Henrikson

**Affiliations:** 1 Department of Oral and Maxillofacial Surgery and Oral Medicine, Faculty of Odontology, Malmö University, Malmö, Sweden; 2 Department of Prosthodontics, Faculty of Odontology, Malmö University, Malmö, Sweden; 3 Department of Orofacial Pain and Jaw Function, Faculty of Odontology, Malmö University, 205 06 Malmö, Sweden; 4 Faculty of Dentistry, National University of Singapore, Singapore, Singapore

**Keywords:** Bruxism, Bite force, Tooth fracture, Veneer porcelain fracture, Health care, Medical research

## Abstract

The aim of this long-term follow-up study was to investigate the relationship between bite force, bruxism, and fractures of teeth and veneer porcelain of fixed dental prostheses. Patients previously assessed as probable bruxers (n = 30) and non-bruxers (n = 21), all rehabilitated with dental implant-supported restorations, underwent a clinical examination and measurement of maximum bite force. A univariate general linear model was used to compare regression lines showing the relationship between fractures and bite force. Bruxers had significantly higher maximum bite force (*p* = 0.023) and higher proportion of tooth/veneer porcelain fractures per total number of tooth/prosthetic units (*p* = 0.045). There was no significant difference in the relationship between frequency of tooth/veneer porcelain fractures and maximum bite force between probable bruxers and non-bruxers (*p* = 0.054). However, there was a significant difference between probable bruxers and non-bruxers when the percentage of fractures in relation to the total number of units was considered instead of the frequency of fractures (*p* = 0.035). Higher maximum bite force in probable bruxers was related to higher prevalence of fractures of teeth and veneer porcelain, emphasizing the potential benefits of pre-treatment assessment of bruxism as well as bite force. Easy-to-use reliable clinical methods for bite force measurement should be tested and implemented in dental practice.

## Introduction

Chipping and fractures of teeth and dental restorations are frequent complications and affect the longevity of teeth and dental prostheses. Chipping refers to the breakage of a small fragment from a brittle material, such as tooth enamel or veneer porcelain, often caused by a force applied near the edge of a tooth or restoration, resulting in a small fracture^1^. These fractures threaten the long-term survival of prosthetic restorations, including both single crowns^2–4^ and more extensive fixed prostheses of longer extension^5–7^. Fractures may lead to the replacement of the prosthetic work, depending on the size and location of the fracture. Regarding natural teeth, enamel and enamel-dentin fractures might also require additional clinical intervention, such as a simple contouring or a direct restoration, depending on the amount of tissue lost^8^.

Various factors have been suggested as possible reasons for the occurrence of fractures in fixed prosthesis and natural teeth in adults, such as sports and vigorous recreational activities^9^, traffic accidents^10^, direct laryngoscopy for tracheal intubation^11^, endodontic treatment^12^, loss of tooth coronal structure concomitantly with presence of extensive direct restorations^13^, sharp edges of enamel without dentin support^14^, and bruxism.

Bruxism—a repetitive masticatory muscle activity often described as grinding or clenching of the teeth—can occur during sleep (sleep bruxism) or while awake (awake bruxism). It has been linked to an increased incidence of technical complications in dental implant-supported prostheses^15^. Therefore, probable bruxism was shown to be a factor in the increase of the prevalence of technical complications in implant-supported prostheses^5,6,15^ and chip off fractures in natural teeth^16^. Probable bruxism can exert significant pressure on dental structures, leading to higher risks of fractures and wear. One of the primary areas of concern with respect to bruxing behavior is the creation of high bite force between the upper and lower dentitions^17^, which can exacerbate damage to both natural teeth and prosthetic components and may necessitate early intervention.

Maximum bite force is a useful indicator of the functional state of the masticatory system and the loading of the teeth^18^. A study observed that the presence of sleep bruxism did not influence the bite force^19^. Moreover, no significant relationship was observed between the occurrence of fracture of implant-supported fixed dental prostheses and the magnitude of bite force^20^. However, bite force during sleep bruxism, although generally being lower^17^, may exceed the amplitude of maximum voluntary bite force during awakeness. This raises the question of whether measures of sleep bruxism activity (e.g. average and peak force, duration and direction of forces) could present a higher risk of damage to natural teeth and fixed dental prostheses than awake bruxism in some individuals. Whereas electromyography provides information on muscle activity, the actual functional output—i.e., bite force—is more accurately assessed using a force transducer.

This study aimed to investigate the relationship between maximum bite force, probable bruxism, and fractures of teeth and veneer porcelain. The null hypothesis of the present study was that a group of probable sleep bruxers will not present higher mean maximum bite force than a group of non-bruxers, and the incidence of prosthesis/teeth fracture will not differ between the groups.

## Methods

### Study sample

A subgroup from 2,670 patients treated with dental implants between 1980 and 2015 at a specialist clinic in Malmö, Sweden, and diagnosed as probable bruxers (n = 98) were reinvited for a clinical examination, together with a matched control group, also treated with dental implants (n = 98). The diagnosis of bruxism for the patients was made in a previous study^15^, on which the present cohort is based. The patients were then clinically re-assessed in the present study. As the aim of this study was to evaluate the long-term consequences of previously identified probable bruxism, the baseline classification was retained to allow assessment of outcomes over time. Both at the time of the diagnosis and for the present study, the patients signed an informed and written consent form agreeing to participate.

Updated personal information about the patients was searched from the data registry of the Swedish Population Registry (*Folkbokföringsregister*) and from the administrative registry of patients of the country region (*PASIS register*, *Region Skåne*).

For patients who gave a telephone number, contact was first attempted by a phone call, with two attempts made within one month. In the case of no response, the patients were sent invitation letters to attend a free clinical consultation at the Faculty of Odontology, Malmö University, Sweden, with an explanation of the aims of the appointment and of the study. The letters were re-sent on three additional occasions, with 4 weeks in between, in case of no response by telephone. The clinical examinations were conducted between April 2022 and March 2024. The study was approved by the Swedish Ethical Review Authority (Dnr 2021–06608-01) and was conducted in accordance with guidelines and regulations including the Declaration of Helsinki (1964).

### Definitions

The assessment of bruxism was based on the patient’s self-report plus the inspection part of a clinical examination to classify which patients are probably bruxers^21^. The recommendations from Lobbezoo et al. have recently been developed into a standardized guideline^22^. The patients’ self-awareness of the condition was evaluated with five questions on bruxism, as proposed in a previous study^23^: (1) Are you aware of the fact that you grind your teeth during sleep? (2) Did anyone tell you that you grind your teeth during sleep? (3) On morning awakening or on awakenings during the night, do you have your jaws thrust or braced? (4) Do you clench your teeth whilst awake? (5) Do you grind your teeth whilst awake? All questions could be answered with either “yes” or “no”. The patients were instructed to answer “yes” if they considered their habit to be frequent enough to be clinically relevant (e.g., frequency of more than thrice a week and/or several hours per day)^23^.

According to the International Classification of Sleep Disorders^24^, the signs and symptoms of bruxism are the (a) presence of regular or frequent tooth grinding sounds occurring during sleep; (b) abnormal tooth wear consistent with above reports of tooth grinding during sleep; (c) transient morning jaw muscle pain or fatigue, and/or temporal headache, and/or jaw locking on awakening consistent with above reports of tooth grinding during sleep. Moreover, the clenching or grinding of the teeth and/or the bracing or thrusting of the mandible during wakefulness was also considered, according to an international consensus report^21^. The clinical examination for assessment of bruxism was carried out by the same trained operator.

The number of units was defined as the total number of teeth and/or fixed prosthetic restorations (either tooth- or implant-supported) in both dental arches. For example, a patient with teeth from first molar to first molar in both arches would have 24 units in total: 12 in the maxilla and 12 in the mandible.

Enamel fractures were defined as those limited to the enamel without exposing dentin or pulp, and enamel-dentin fractures were defined as tooth fractures with loss of enamel and dentin without exposing the dental pulp^25^.

Two calibrated examiners (TB, SS) performed the clinical assessment of number of tooth/prosthesis units and the presence of fractures. This clinical examination was performed before the maximum bite force assessment and the examiners were blinded to the patients’ group allocation as non-bruxers or probable bruxers.

### Data collection

The data collected consisted of patient’s sex, patient age at the clinical examination, smoking habits (yes, no, former smoker), number of tooth/prosthesis units, number of tooth/veneer porcelain fractures, and maximum bite force.

### Maximum bite force

**Fig. 1:**
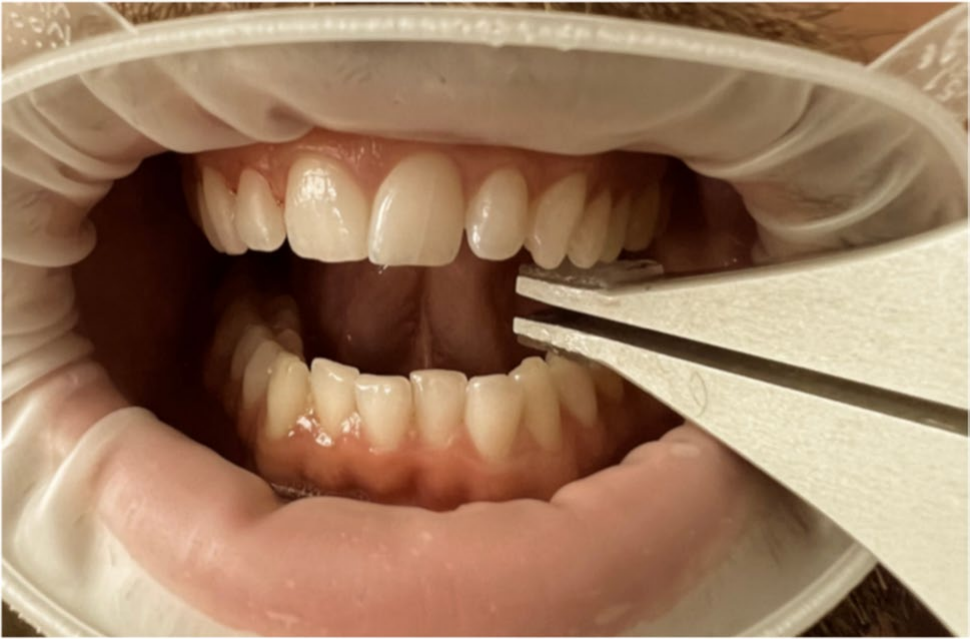
Unilateral measurement of maximum biteforce in the premolar region with a previously used customized protocol^26^.

Maximum bite force was evaluated by a separate examiner (NS), blinded to group allocation and to the number of fractures registered in the clinical examination. The measurements were performed in the premolar region at the right side with a previously used customized protocol^26^ conducted with a strain gauge-based bite force transducer (Umeå University, Physiology Section, Umeå, Sweden) that displays the maximum force in Newton (N) (Fig. 1). This custom-made transducer has been used in several previous experimental studies involving bite force assessment and is designed for unilateral recordings with high sensitivity to force variations. Calibration was performed using known loads to ensure measurement consistency^26–28^. The patients were asked to bite as hard as possible until they felt that they had achieved their maximum bite force (2 to 5 s). The bite force meter, using a digital display not visible to the patient, provided the maximum force in Newton (N) applied per bite application. All measurements were conducted in a single session, with a 30-s rest in between, and the average from these three measurements was calculated.

#### Statistical methods

Mean, standard deviation, and percentages were presented as descriptive statistics. The Kolmogorov–Smirnov test was performed to evaluate the normal distribution of the variables, and Levene’s test evaluated homoscedasticity. The performed tests for the two independent groups were Student’s *t*-test or Mann–Whitney test, depending on the normality. Pearson’s chi-squared test or Fisher’s exact test was used in the analysis of contingency tables of categorical data.

Scatter plots were drawn to describe how the frequency of fractures of tooth structure and/or veneer porcelain of fixed prostheses varied in relation to the maximum bite force values of the patients, for which a linear regression model was presented. A univariate general linear model was used to compare the regression lines of the “fractures/bite force” relationship between non-bruxers and probable bruxers. The degree of statistical significance was considered *p* < 0.05. All data were statistically analyzed using the Statistical Package for the Social Sciences (SPSS) version 29 software (SPSS Inc., Chicago, IL, USA).

## Results

Data from the national population register showed that out of 196 patients examined 2015 in the original prospective study cohort, 101 individuals were either deceased or no longer registered as Swedish citizens. This left a cohort of 95 patients eligible for participation. Of these, 51 patients (54%) were examined with both clinical examination and laboratory tests in the long-term follow-up. An additional 28 patients were listed in at least one of the registries but did not reply to the letters or phone calls.

In total, 53 participants were examined, although one was found to be completely edentulous, and another was confirmed by their physician to have cognitive impairment. Data from 51 patients were ultimately eligible for clinical evaluation, of which, 30 had been previously classified as probable bruxers and 21 as non-bruxers. Table 1 presents the comparative data between the groups.

**Table 1:** Demographic data, maximum bite force, and number of tooth/prosthesis units and fractures.

	Probable bruxers	Non-bruxers	*p* value
N	30	21	
Men/women	12/18	10/11	0.589 ^a^
Age, in years, mean ± SD (min, max)	60.5 ± 17.4 (30.9, 92.2)	64.9 ± 16.0 (31.8, 86.1)	0.379 ^b^
*Smoking, n (%)*			
Yes	7 (23.3)	1 (4.8)	0.187 ^a^
Former	8 (26.7)	8 (38.1)	
No	15 (50.0)	12 (57.1)	
Maximum bite force, in Newtons, mean ± SD (min, max)	618 ± 199 (232, 910)	486 ± 197 (153, 808)	0.023 ^c^
Number of tooth/prosthesis units, mean ± SD (min, max)	26.3 ± 3.3 (18, 32)	26.1 ± 4.2 (19, 32)	0.859 ^c^
Number of tooth/prosthesis fractures, mean ± SD (min, max)	1.8 ± 1.9 (0, 6)	1.0 ± 1.4 (0, 5)	0.055 ^b^
Fractures/units *	7.3 ± 7.6 (0, 25.0)	3.5 ± 5.1 (0, 15.6)	0.045 ^b^

*SD* standard deviation.

* Percentage of the number of tooth/veneer porcelain fractures in relation to the total number of tooth/prosthesis units.

^a^ Pearson’s chi-squared test.

^b^ Mann–Whitney test.

^c^ Student’s *t*-test.

Six out of the 51 patients presented shortened dental arch, two women and four men (*p* = 0.383; Fisher’s exact test). The mean maximum bite force did not differ significantly between men and women (*p* = 0.482; Mann–Whitney test) or with the variation of age of the patients (*p* = 0.785; linear regression). There was a significant reduction in the number of tooth/prosthesis units with increasing age of the patients (*p* < 0.001; linear regression). The number of tooth/prosthesis units varied inversely with the maximum bite force, *i.e.*, the greater number of tooth/prosthesis units, the smaller the maximum bite force (*p* = 0.017; linear regression).

**Fig. 2:**
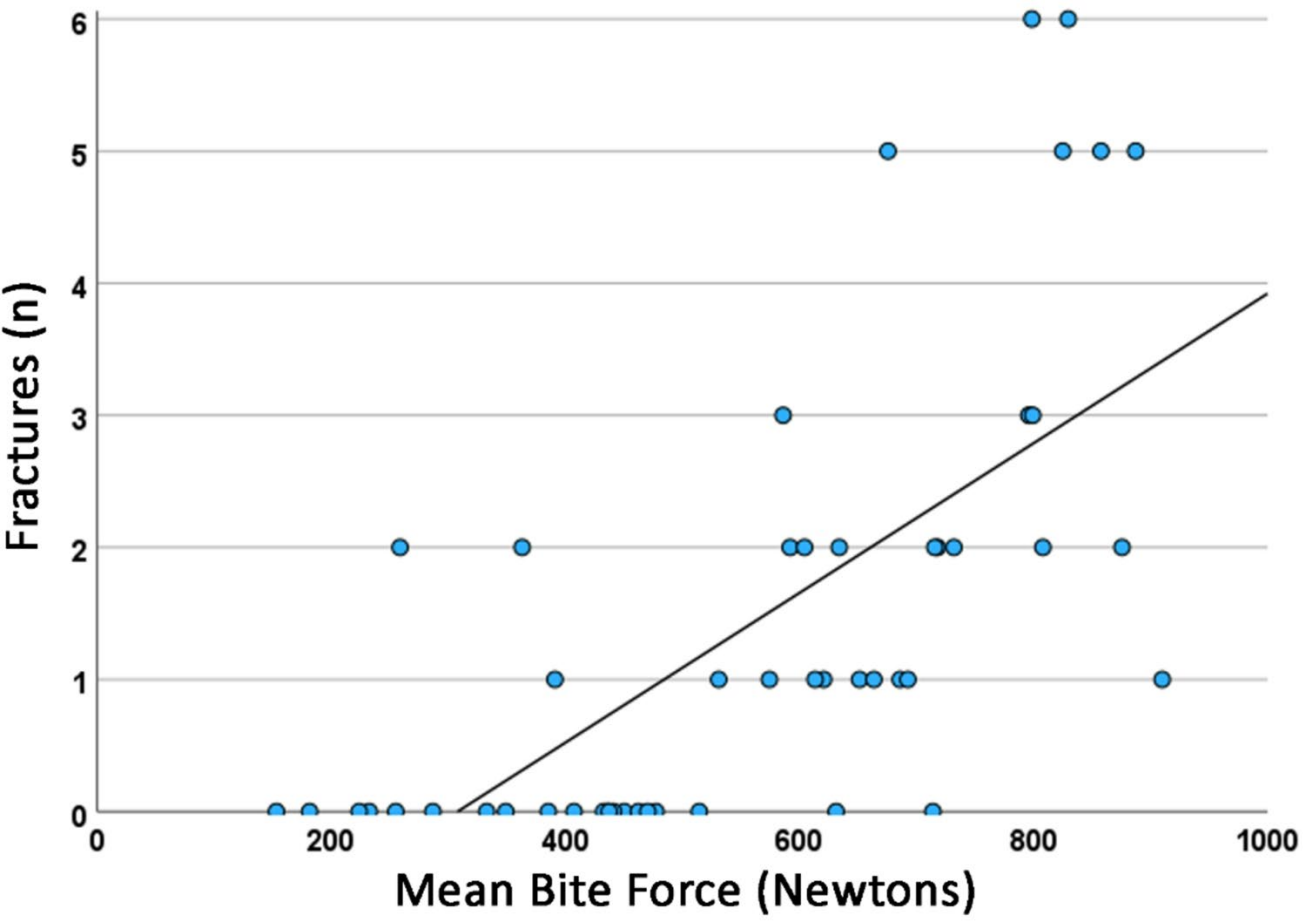
Relationship of the total number of tooth/veneer porcelain fractures in function of the maximum bite force in Newtons. The continuous line represents the regression line (*r*^2^ = 0.457).

**Fig. 3:**
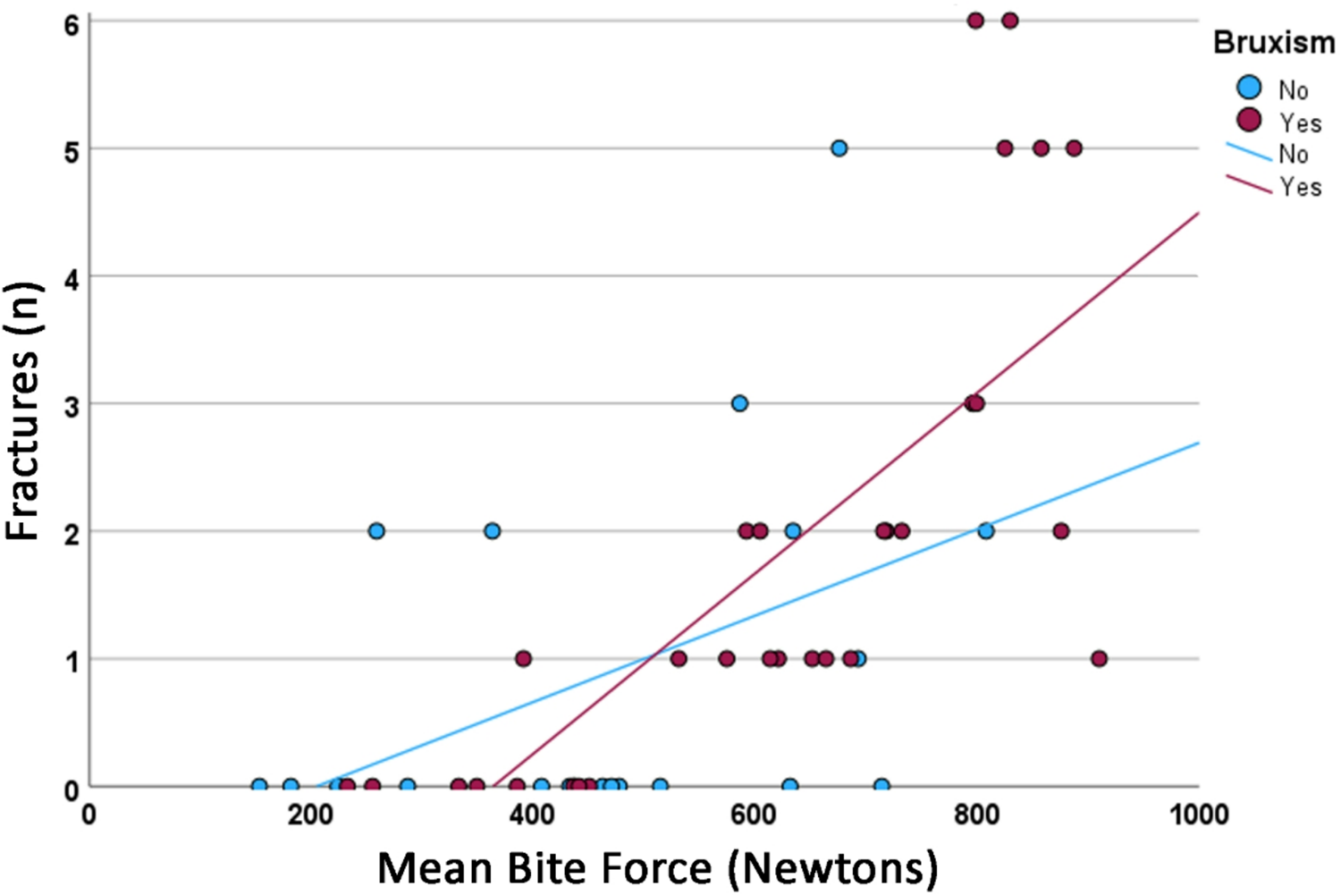
Comparison of the relationship of the total number of tooth/veneer porcelain fractures in function of the maximum bite force in Newtons between probable bruxers (purple dots) and non-bruxers (blue dots). The continuous lines represent the regression lines (probable bruxers, *r*^2^ = 0.570; non-bruxers, *r*^2^ = 0.217).

The relationship of the frequency of fractures of tooth structure and/or veneer porcelain of fixed prostheses in function of the mean maximum bite force in Newtons is shown in Fig. 2. The linear regression coefficient (*r*^2^) for this model was 0.457. In Fig. 3, this relationship is compared between the groups of probable bruxers (*r*^2^ = 0.570) and non-bruxers (*r*^2^ = 0.217). The difference between the regression lines was not statistically significant (*p* = 0.054).

The equations for the corresponding regression line of the groups were:

$${\text{Probable bruxers}}:{\text{ y}} = - {2}.{57 } + { 7}.0{7}*{1}0^{{ - {3}}} .{\text{x}}$$

$${\text{Non}} - {\text{bruxers}}:{\text{y}} = - 0.{69 } + { 3}.{38}*{1}0^{{ - {3}}} .{\text{x}}$$

**Fig. 4:**
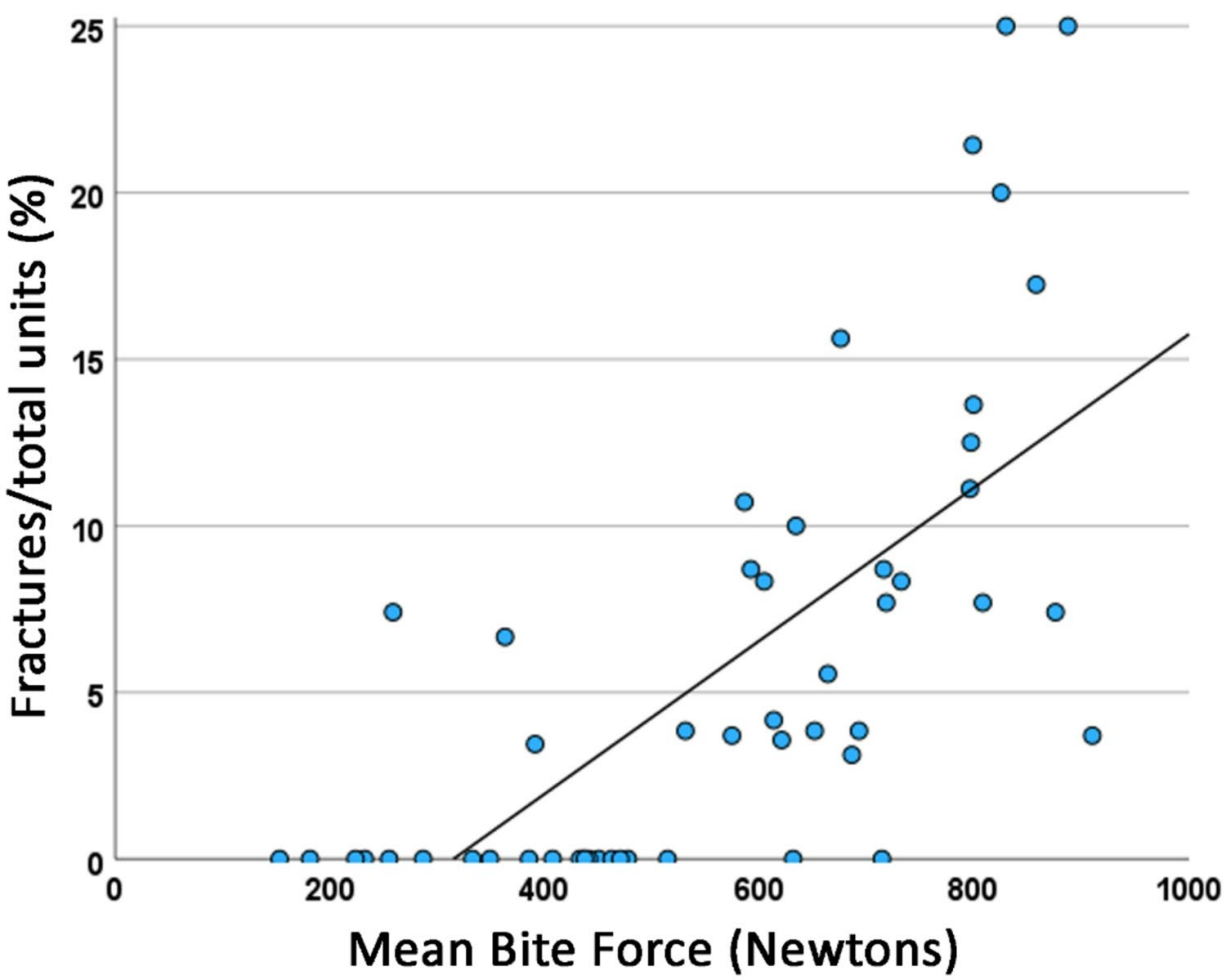
Relationship of the percentage of the number of tooth/veneer porcelain fractures in relation to the total number of tooth/prosthesis units in function of the maximum bite force in Newtons. The continuous line represents the regression line (*r*^2^ = 0.477).

**Fig. 5:**
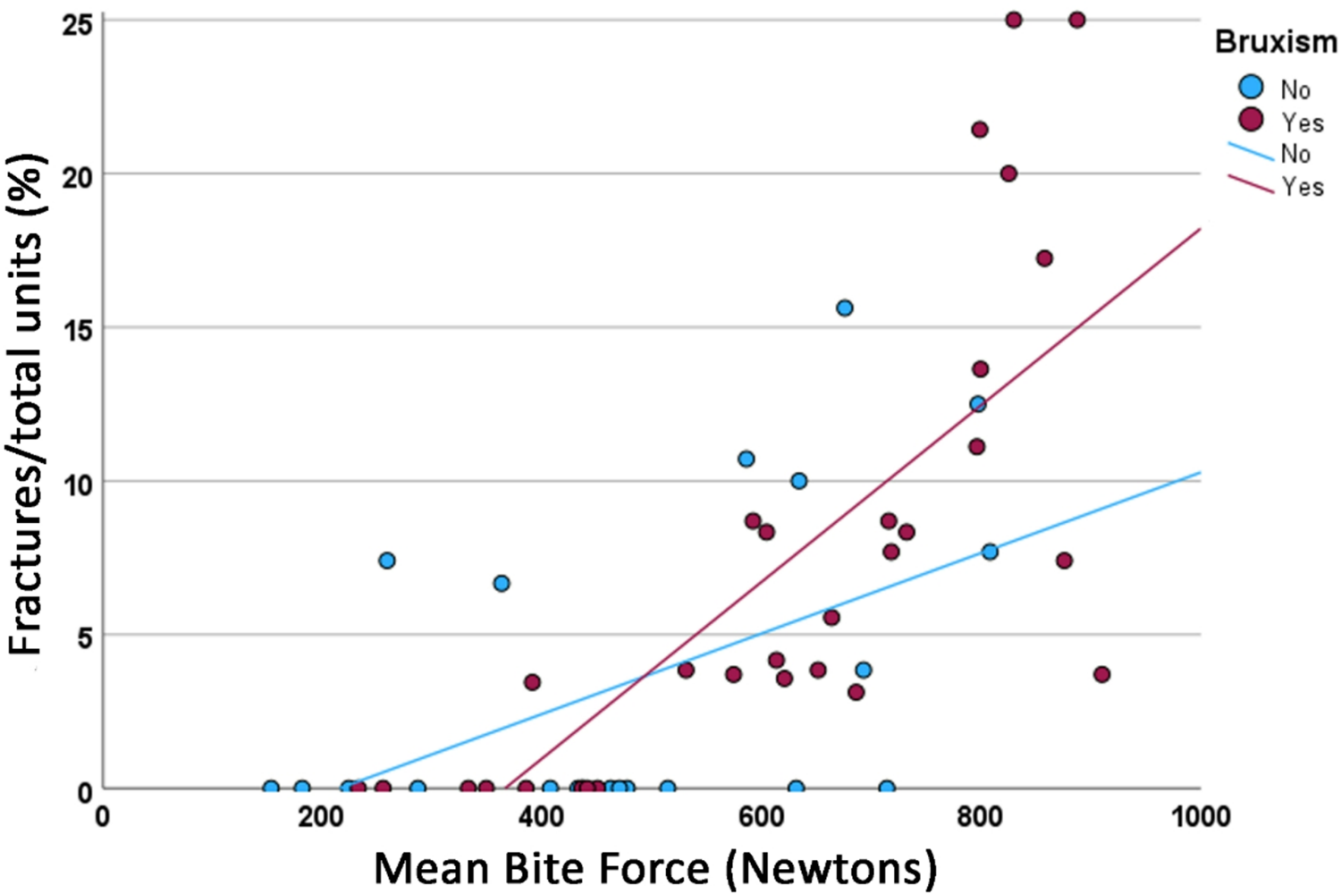
Comparison of the relationship of the percentage of the number of tooth/veneer porcelain fractures in relation to the total number of tooth/prosthesis units in function of the maximum bite force in Newtons between probable bruxers (purple dots) and non-bruxers (blue dots). The continuous lines represent the regression lines (probable bruxers, *r*^2^ = 0.564; non-bruxers, *r*^2^ = 0.254).

Figure 4 shows the relationship between the percentage of the number of tooth/veneer porcelain fractures in relation to the total number of tooth/prosthesis units in function of the mean maximum bite force in Newtons (*r*^2^ = 0.477), while in Fig. 5, this relationship is compared between the groups of probable bruxers (*r*^2^ = 0.564) and non-bruxers (*r*^2^ = 0.254). The difference between the regression lines was statistically significant (*p* = 0.035).

## Discussion

The main aim of the present study was to investigate the relationship between maximum bite force, probable bruxism, and fractures of teeth and veneer porcelain. The null hypothesis tested that there would be no difference in mean maximum bite force between probable bruxers and non-bruxers, and that the incidence of prosthesis/teeth fracture would not differ between the groups. According to the results, the difference of the regression lines inclination (slopes) for the relationship of the frequency of fractures of tooth structure and/or veneer porcelain of fixed prostheses in function of the mean maximum bite force between probable bruxers and non-bruxers was not statistically significant. However, the slopes were significantly different when the percentage of fractures in relation to the total number of units was considered.

Here lies the issue of either considering the frequency of fractures, regardless of total number of teeth/prostheses, or the percentage of teeth that were fractured. This is important since two studies observed that the number of natural teeth varies closely with the bite force, *i.e.*, the more natural teeth one has, the greater their bite force^29,30^. However, it was observed in an experimental in vitro and in vivo study with simulations of short dental arches that the tooth-related bite force in premolars and anterior teeth increased with missing molar support^31^, suggesting that individuals with short dental arches may develop stronger habitual bite forces to improve chewing efficacy, as they have less teeth^32^. Nevertheless, only six patients in the present study presented short dental arches, with most patients showing intercalated tooth loss in a long dental arch.

The present results suggest that probable bruxers present a statistically significant higher mean maximum bite force than non-bruxers. This aligns with some studies^33^ but contrasts with others^34,35^ which found no influence of bruxism on maximum bite force values. Helkimo and Ingervall^36^ found that bruxers present a higher bite force only in the incisor region, but not between the molars. However, an investigation showed a significant correlation between frequency, peak amplitude, or duration of bruxism events with maximum nocturnal bite force, suggesting that severe bruxism patients may have a strong nocturnal bite force^17^.

As excessive muscle contraction is generally inhibited by the consciousness of the central nervous system^37^. In a study that compared bite force during varying submaximal loads between bruxers and non-bruxers^38^ it was observed that the patients with bruxism used significantly higher bite forces to hold each given submaximal load compared to the non-bruxers subjects. These studies^17,33,38^ add evidence to support the present results, despite divergent results from other investigations^34,35^. Regarding the consequences of bruxism, in the present study assessing the frequency of fractures, it is reasonable to assume that bruxers display more episodes of tooth contact, which may cause more stress on teeth and dental restorations, thereby resulting in more fractures. This is supported by studies utilizing ecological momentary assessment that show a higher frequency of tooth contact in individuals with possible/probable bruxism^39^. Nevertheless, it seems important not only to consider the frequency of the jaw muscle contractions but also the magnitude of the contractions and the duration of such activities, i.e., a more composite measure of the net load on the dental tissues and restorations^40,41^.

However, considering age, a significant difference of maximum bite force with age was not observed in the present study, in agreement with some other studies^29,35^. However, in a different study^30^, the values found for the bite force decreased with increasing age, particularly for women. Helkimo et al.^30^ suggested that most of this reduction with increasing age was probably due to the age-dependent deterioration of the dentition. Indeed, the number of tooth/prosthesis units was significantly reduced with increasing age. A possible explanation for the lack of significantly different maximum bite force with age in the present study, despite the reduction in the number of tooth/prosthesis units, could be related to the composition of our study sample, with predominately older participants, or to the measurement of the bite force only in the premolar region. In Helkimo et al.’s^30^ study, the apparatus for measuring the maximal bite force was used both in the area of the first molars and in the area of the incisors. However, when the molars were missing, the recordings were made in the area of the premolars or the canines. Therefore, the patients who had lost their molars, supposedly the elder patients, since the loss of posterior occlusal support increases with age^42^, had their measurements taken in more anterior areas of the dentition, resulting in lower mean values for the maximum bite force. As shown in the same study^30^, the mean bite force was smaller when the bite fork was placed in a more anterior region of the dentition. Moreover, Helkimo et al.^30^ also included denture wearers in study, which was not the case here. The probability of wearing a complete removable denture increases with age^44^, although the difference in mean age between patients with natural dentition and patients with complete dentures was not reported in the study of Helkimo et al.^30^. We also expect that the patients evaluated in the present study generally had more natural teeth as well as fixed dental prosthesis, as opposed to removable dentures, in line with the general improvement of oral health in the population over the last decades.

Moreover, the present results did not reveal a significant sex-related difference in the maximum bite force, which is at odds with the higher values for men found in several other studies, although several of these studies were performed in young adults and not in prosthetically rehabilitated patients, often applying strict criteria regarding the number of teeth and overall dentition status^29,34,35,45–47^. The higher maximum bite force in men was hypothesized to be related to the fact that women have smaller teeth, which corresponds to a smaller periodontal ligament area and consequently might deliver a lower bite force than seen in men^35^. Another possible explanation, more commonly accepted, could be the greater muscular potential of males, which may be attributed to the anatomic differences between different sexes^48^. In this context, the masseter muscles of men have muscle fibers with a larger diameter and a greater sectional area than those of women^46^. The exact explanation for this divergence between the present results and other studies is, however, unclear but could be related to the relatively small sample size and differences in terms of prosthetic units between men and women in our patient sample. However, the focus for the present study was not possible sex differences but the relationship between bite force, bruxism and fractures.

The present study is not without limitations. First, the patients may have had some concerns about causing damage or generating pain and tenderness in the teeth, supportive structures, temporomandibular joint, or masticatory muscles during the measurements, which may underestimate the values of bite force^18^. Moreover, the reliability of bite force measurements may be influenced by other individual factors that may affect the measurements, including craniofacial morphology, state of dentition and occlusion, vertical separation of teeth and jaws (vertical dimension), strength of jaw closing muscles, functional disturbances of the masticatory system such as the presence of pain and temporomandibular disorders, pain or discomfort thresholds of each subject, mental state during the experiment, and attitude of the investigator^49–55^. In addition to keeping the bite force sensor/transducer in the same place in the dental arch for all participants, namely, in the premolar area, since a greater bite force is expected as the bite point moves posteriorly^56^, other factors that may also influence the measurements that were analyzed here include the sex and age of the individuals. The measurements were conducted only on the right side of the dentition, but this is not seen as an issue, as studies show that individuals have approximately equal bite force on the left and right side^19,51^. Future studies may consider adding direct bite force recordings with digital occlusal analysis systems, such as T-Scan, or indirect assessment with electromyography, to provide a more comprehensive assessment of occlusal loading patterns and force magnitude and distribution.

It is also worth noting that 15 participants reported using an occlusal splint during the follow-up. Although splints are commonly used to manage sleep bruxism, they are rarely used daytime. Since awake bruxism, may play an important and sometimes an overlooked role in the development of tooth and restoration fractures, splint use in these cases likely had a limited effect on preventing damage. Furthermore, for assessment of awake bruxism, the recent development of smartphone-based applications such as BruxApp^56^ for Ecological Momentary Assessment (EMA) of awake bruxism can provide a more comprehensive evaluation of daytime parafunctions in future studies.

## Conclusion

Higher mean maximum bite force in probable bruxers is associated with an increased prevalence of fractures of teeth and veneer porcelain fixed prosthesis. It may therefore be of value to assess bruxism and to apply reliable but easy-to-use clinical methods for the measurement of bite force when planning dental prosthetic treatment.

## Data Availability

The data supporting the findings of this study are available from the corresponding author upon reasonable request. Due to ethical considerations, access to the data is subject to restrictions.
